# Muscle-Specific Myosin Heavy Chain Shifts in Response to a Long-Term High Fat/High Sugar Diet and Resveratrol Treatment in Nonhuman Primates

**DOI:** 10.3389/fphys.2016.00077

**Published:** 2016-03-02

**Authors:** Jon-Philippe K. Hyatt, Lisa Nguyen, Allison E. Hall, Ashley M. Huber, Jessica C. Kocan, Julie A. Mattison, Rafael de Cabo, Jeannine R. LaRocque, Robert J. Talmadge

**Affiliations:** ^1^Department of Human Science, Georgetown UniversityWashington, DC, USA; ^2^Department of Biological Sciences, California State Polytechnic UniversityPomona, CA, USA; ^3^Translational Gerontology Branch, National Institute on Aging, National Institutes of HealthBaltimore, MD, USA

**Keywords:** skeletal muscle, MHC, rhesus macaque, PGC-1α, GLUT4

## Abstract

Shifts in myosin heavy chain (MHC) expression within skeletal muscle can be induced by a host of stimuli including, but not limited to, physical activity, alterations in neural activity, aging, and diet or obesity. Here, we hypothesized that both age and a long-term (2 year) high fat/high sugar diet (HFS) would induce a slow to fast MHC shift within the plantaris, soleus, and extensor digitorum longus (EDL) muscles from rhesus monkeys. Furthermore, we tested whether supplementation with resveratrol, a naturally occurring compound that has been attributed with augmenting aerobic potential through mitochondrial proliferation, would counteract any diet-induced MHC changes by promoting a fast to slow isoform switch. In general, we found that MHC isoforms were not altered by aging during mid-life. The HFS diet had the largest impact within the soleus muscle where the greatest slow to fast isoform shifts were observed in both mRNA and protein indicators. As expected, long-term resveratrol treatment counteracted, or blunted, these diet-induced shifts within the soleus muscle. The plantaris muscle also demonstrated a fast-to-slow phenotypic response to resveratrol treatment. In conclusion, diet or resveratrol treatment impacts skeletal muscle phenotype in a muscle-specific manner and resveratrol supplementation may be one approach for promoting the fatigue-resistant MHC (type I) isoform especially if its expression is blunted as a result of a long-term high fat/sugar diet.

## Introduction

Skeletal muscle phenotype is determined, in part, by the type of myosin heavy chain (MHC) protein expressed throughout the tissue. The four predominant MHC genes expressed in adult skeletal muscle (types I, IIa, IIx, and IIb) are associated with correspondingly named fiber types (I, IIA, IIX, and IIB). The fiber types can be generally categorized from contractile speed and aerobic or anaerobic characteristics. Relative to fast muscles (with a preponderance of type II fibers), slow (type I) muscles are high in capillary density and mitochondrial volume, contain a greater proportion of aerobic enzymes, and are generally fatigue resistant. Oxidative muscles also have a greater sensitivity to insulin when compared to fast muscle phenotypes (Koerker et al., 1990). Of the fast fiber types, type IIa has greater oxidative characteristics than types IIx or IIb. In fact, recent work has shown that fast isoforms can have different oxidative capacities across different muscles (Bloemberg and Quadrilatero, 2012). In humans, there are a number of internal or external variables including physical activity (Klitgaard et al., 1990; Minetto et al., 2013; Mosole et al., 2014) or inactivity (Borina et al., 2010), changes in neural stimulus (Mu et al., 2012), aging (Klitgaard et al., 1990; Vandervoort, 2002; D'Antona et al., 2003; Short et al., 2005; Mosole et al., 2014), and diet or obesity (Krotkiewski and Björntorp, 1986; Lillioja et al., 1987; Hickey et al., 1995; Kriketos et al., 1996; Tanner et al., 2002; Matsakas and Patel, 2009) that can influence MHC isoform expression. For example, an age-related slow to fast MHC isoform shift has been reported (D'Antona et al., 2003) although others have observed the opposite effect, e.g., fast to slow, effect (Short et al., 2005). In addition, a high fat diet has been shown to reduce the percentage of fibers expressing type I MHC in rodent and human muscles, suggesting a reduction in overall oxidative capacity, insulin sensitivity, and glucose tolerance (Lillioja et al., 1987; Abou Mrad et al., 1992; Hickey et al., 1995; Kriketos et al., 1996).

In recent years, resveratrol (3,5,4′-trihydroxytransstilbene) supplementation has been investigated as a possible strategy to counteract the detrimental impact of physical inactivity- or high fat diet-associated conditions. Resveratrol is a common polyphenolic compound found in the skins of red grapes and in other plant products that has been linked to enhanced longevity in lower organisms (Viswanathan et al., 2005; Valenzano et al., 2006) and mice (Baur et al., 2006) through activation of the sirtuin-1 (Sirt1) pathway. Resveratrol supplementation has been shown to be beneficial against atherosclerosis (Borriello et al., 2010), cerebral ischemia (Simão et al., 2012), and type II diabetes (Borriello et al., 2010). Resveratrol alters the progression of type II diabetes, for example, by enhancing insulin sensitivity within adipose tissue (Jimenez-Gomez et al., 2013) and skeletal muscle (Lagouge et al., 2006). Additionally, resveratrol has been shown to augment mitochondrial biogenesis in endothelial cells (Csiszar et al., 2009), brown adipose tissue (Lagouge et al., 2006), and skeletal muscle (Lagouge et al., 2006). In fact, 15 weeks of resveratrol supplementation (400 mg/kg/day) in mice on a high-fat diet induced changes within skeletal muscle that paralleled effects attributed to aerobic exercise training including an increase in citrate synthase activity, mitochondrial density, and maximal oxygen consumption, e.g., VO_2max_ (Lagouge et al., 2006). Resveratrol consumption appeared to impact the aerobic capacity of skeletal muscle through the activation of the Sirt1-PGC-1α pathway and an enhancement of mitochondrial density (Lagouge et al., 2006). In contrast, however, recent epidemiological evidence suggests that resveratrol does not positively influence changes in inflammation, cardiovascular disease, cancer, or longevity in humans (Semba et al., 2014).

The notion that resveratrol supplementation may improve resistance to age- and diet-related conditions or diseases through metabolic enhancements is largely supported (Barger, 2013; Raederstorff et al., 2013), although recent findings highlight an additive physiological effect when resveratrol is combined with aerobic activity (Dolinsky et al., 2012). However, there is less evidence relating these metabolic changes (Lagouge et al., 2006) with concomitant muscle fiber phenotypic, e.g., MHC, shifts within skeletal muscle following resveratrol treatment. Initial reports suggest that resveratrol supplementation (Ljubicic et al., 2014), or enhanced expression of PGC-1α (Selsby et al., 2012), promotes a fast to slow MHC isoform shift in dystrophic *mdx* mice. Analysis of swine skeletal muscle following 49 days of resveratrol treatment indicated an increase specifically in MHC IIa mRNA and a reduction in glycolytic enzyme activity (Zhang et al., 2015).

In the present study, the age-, diet-, and resveratrol-related implications on skeletal muscle were investigated with a specific focus on muscle fiber phenotypic changes occurring with long-term (2 year) resveratrol treatment. It was hypothesized that age and a high fat/high sugar diet would induce a slow to fast MHC shift and it was expected that resveratrol supplementation would counteract these phenotypic shifts by promoting a fast to slow isoform switch. To assess the muscle-specific changes with age, diet, and resveratrol, mRNA and protein MHC profiles were performed in three distinct muscles of rhesus monkeys, including the predominantly slow soleus muscle and mixed extensor digitorum longus (EDL) and plantaris (PLT) muscles. In general, we show that MHC isoform shifts are not heavily influenced by aging during mid-life and that a long-term high fat/sugar diet induces a slow to fast isoform shift in a muscle-specific manner. Additionally, resveratrol treatment drives a fast to slow MHC phenotypic shift also in a muscle-specific fashion. Specifically, we show that the postural soleus muscle is the most sensitive to changes in diet or resveratrol treatment.

## Material and methods

### Animals

Twenty-four adult male rhesus monkeys (*Macaca mulatta*) were housed continuously at the National Institutes of Health (NIH) Animal Center (Poolesville, MD). All experimental and animal care procedures were approved by the Animal Care and Use Committee of the National Institute on Aging Intramural Research Program and are described in detail elsewhere (Fiori et al., 2013; Jimenez-Gomez et al., 2013; Mattison et al., 2014). It should be noted that the present study was a part of a larger project entitled “Effects of resveratrol with a high fat and sugar diet in monkeys” which aimed to elucidate the long term impacts of resveratrol supplementation on overall health. To date, several reports pertaining to cardiovascular physiology (Mattison et al., 2014), inflammation and immunology (Jimenez-Gomez et al., 2013), and insulin action and signaling (Fiori et al., 2013; Jimenez-Gomez et al., 2013) have resulted from this project. The scope, aim, and hypotheses tested in the present study were generated post hoc through collaboration.

### Diet and resveratrol supplementation

After a baseline assessment period, the animals were randomized into one of three groups (Fiori et al., 2013; Jimenez-Gomez et al., 2013): age-matched control monkeys were placed on healthy standard diet (*n* = 4) and compared to monkeys placed on a high-fat/high-sugar diet in combination with either placebo (HFS; *n* = 8) or resveratrol (HFSR; *n* = 7). A young control group (*n* = 5) was added for comparison but was not part of the original longitudinal resveratrol monkey cohorts. The control animals received a purified diet consisting of 18.2, 13.1, and 68.7% of kcal from protein, fat, carbohydrate, respectively; approximately 2.2% of this diet consisted of sucrose by weight. During baseline periods, the HFS and HFSR animals received the control diet and were gradually moved to the HFS diet (15.8, 42.3, 41.9% of kcals from protein, fat, and carbohydrate, respectively; 27% sucrose by weight; Teklad; Harlan, Indianapolis, IN) over a 3-week period. Monkeys received two meals per day at estimated *ad libitum* levels throughout the study. Water was available *ad libitum*. The average food consumption for weekly periods was the same for all three groups.

Resveratrol was supplied by DSM Nutritional Products North America (Parsippany, NJ). Dosages were determined based on earlier work denoting the protective dose in a rodent model (Baur et al., 2006). Equivalent dosages for monkeys in the present study were calculated using an average monkey body weight of 12.1 kg. For the first year, HSFR monkeys received a total dose of 80 mg/day (40 mg/meal); during the second year, 480 mg/day (240 mg/meal) was provided. The resveratrol was incorporated into a cherry-flavored primate treat (Bio-Serv, Frenchtown, NJ) that was given to the monkeys prior to each meal. Non-resveratrol control and HFS animals received a cherry-flavored placebo treat (PRIMA-Treats® Bio-Serv).

### Experiment termination and tissue collection

At 24 months, the monkeys were deeply anesthetized with a lethal dose of sodium pentobarbital (50 mg/kg, intraperitoneal). Once sedated, each monkey was perfused with cold lactated Ringer's solution until death. The soleus (Sol), plantaris (Plt), and EDL muscles were removed from the hindlimbs, trimmed, and cleaned of excess connective tissue, weighed, and flash frozen in liquid nitrogen. Control animal tissues were segregated based on age into young (Con-Y; *n* = 5) and old (Con-O; *n* = 4) groups. Whole muscles were stored at −80° C until further analysis. Serum collected at experiment termination indicated that the concentrations of resveratrol and resveratrol-3-O-sulfate within HFSR animals were 27.7 ± 8.6 and 239.1 ± 82.2 ng/mL, respectively (Fiori et al., 2013).

### RNA extraction and real-time PCR analysis

A representative cross-sectional sample of tissue was removed from the belly of each muscle and total RNA was extracted from muscle homogenates using TRI Reagent according to the manufacturer's protocol (Molecular Research Center, Cincinnati, OH). cDNA was then synthesized from 1 μg of total RNA from each sample using Superscript II according to the manufacturer's instructions (Life Technologies, Carlsbad, CA). For expression analysis, 1 μL of the reverse transcription reaction mixture containing the cDNA was used for real-time PCR of PGC-1α and MHC types I, IIa, and IIx. For MHC type IIb expression, 2 μL of cDNA was used.

Primers for quantitative real time PCR (qPCR) were designed using Primer3 Input version 0.4.0. (Untergasser et al., 2012) and the *M. mulatta* genome sequence (Baylor College of Medicine Human Genome Sequencing Center). Reverse primer sequences were determined using the highly diverged 3′UTR sequence to ensure myosin isoform specificity. Primer sequences for MHC and PGC-1α amplification are shown in Table 1. Ribosome protein L13a (RPL13A) was chosen as the internal housekeeping gene (Table 1) based on previous work in rhesus monkey tissue (Ahn et al., 2008; Noriega et al., 2010). To each qPCR reaction, 100 nM of either MHC type I, IIa, IIx, IIb, PGC-1a, or RPL13A primer pair was added. Amplification and real-time PCR curves for each sample was performed using the ABI7900HT sequence detection system (Life Technologies) in the presence of 1X standard SYBR green (Life Technologies) using the following parameters: denaturing steps at 50° C for 2 min and 95° C for 10 min, followed by 95° C for 15 s, and 60° C for 1 min for 40 cycles. For each sample, qPCR reactions were performed in duplicate, averaged, and normalized by subtracting the corresponding RPL13A threshold cycle (ΔC_T_). The relative abundance of transcripts for MHC isoforms and PGC-1α was then calculated using the ΔΔC_T_ comparative method (Livak and Schmittgen, 2001). To determine the relative expression percentage for each MHC gene, the relative abundance values for each MHC isoform in a given muscle sample were added together and the percent expression of each isoform was calculated using this summed value.

**Table 1:** **Primer sequences used for quantitative real-time PCR analysis**.

**Common name**	**Gene**	**Primer sequences (5′–3′)**		**Expected size (bp)**	
				**PCR**	**qRT-PCR**
MHC Type I	MYH7	GCTGCAGCTAAAGGTCAAGGCC	forward	1179	220
		GCAGATCAAGATGTGGCAAAGC	reverse		
MHC Type IIa	MYH2	GGTAGATAAACTTCAGGCAAAAGTG	forward	1881	221
		TCCATGGCATCAGGACGTGG	reverse		
MHC Type IIx	MYH1	TGGACAAATTGCAAGCAAAG	forward	2097	234
		GTGCATTTCTTTGGTCACC	reverse		
MHC Type IIb	MYH4	GCAGGACTTGGTGGACAAAT	forward	1313	239
		CATTTTCTTCCATTAGAATG	reverse		
PGC-1α	PPARGC1A	GCTGACAGATGGAGACGTGA	forward	2146	178
		TGCATGGTTCTGGGTACTGA	reverse		
Ribosome protein L13a	RPL13A	CAAGGTGTTTGACGGCATCC	forward	677	193
		GATCTTGGCTTTCTCCTTCCTCTT	reverse		

*MHC, myosin heavy chain; primer for the RPL 13A gene was constructed based on previous work (Ahn et al., 2008; Noriega et al., 2010)*.

### Total protein isolation, MHC isoform separation, and MHC IIa western blot analysis

Muscle samples were prepared using the method described by Talmadge and Roy (1993). Briefly, frozen muscle cross-sectional samples were minced with scissors in 2 volumes of ice-cold radioimmunoprecipitation assay (RIPA) buffer consisting of 150 mM sodium chloride, 1% triton X-100, 1% sodium deoxycholate, 0.1% SDS, 50 mM Tris-HCL, pH 7.5, 2 mM EDTA at pH 8.0 with protease and 1:100 volume Halt ™ phosphatase and protease inhibitor cocktail added (100 mM ABESF-HCl, 80 μM Aprotinin, 5 mM Bestatin, 1.5 mM E-64, 0.5 M EDTA, 2 mM Leupeptin, 1 mM, and Pepstatin A). Homogenates were centrifuged at 10,000 g for 10 min at 4° C and the pellets were resuspended in fresh RIPA buffer. Total protein quantification was completed using the Bradford technique with bovine serum albumin as the standard. Samples were diluted to a final concentration of 0.00833 μg/μl in water and 2x buffer containing SDS, glycerol, β-mercaptoethanol, bromophenol blue, and Tris-HCL.

**Figure 1:**
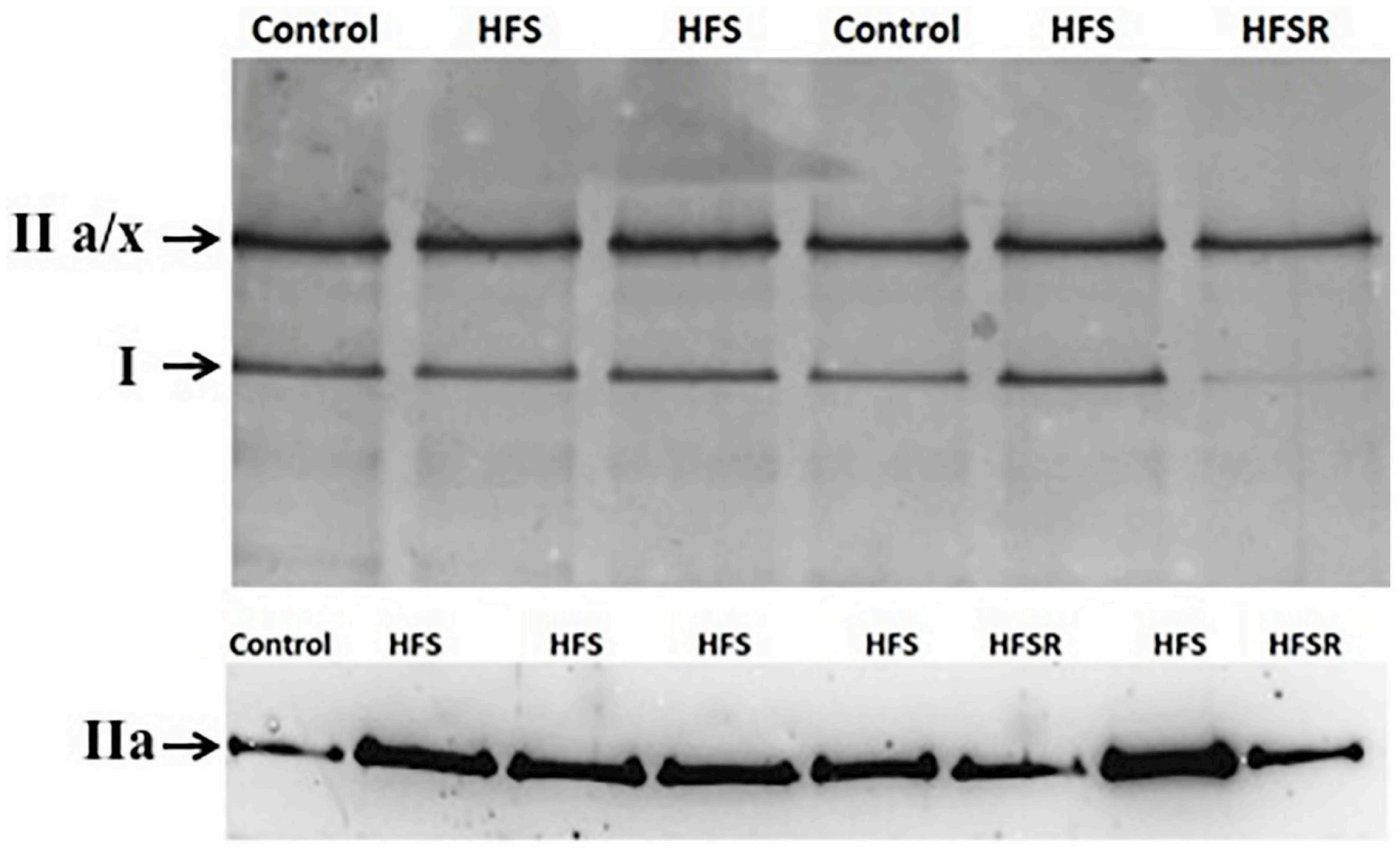
**Rhesus macaque MHC protein isoforms identified by SDS-PAGE (*top*) and western analysis (*bottom*)**. Total MHC protein samples were separated using 8.5% gels that produced two principal bands: type I and IIa/x. To determine the relative contribution of MHC type IIa within each sample, blots were probed using the SC-71 antibody generously supplied by S. Schiaffino (University of Padova, Padova, Italy). Representative images were produced using plantaris muscle samples. HFS, high fat/high sugar diet group; HFSR, HFS diet with resveratrol supplementation.

To determine the percent expression of MHC isoforms in each muscle, samples were run on 8.5% SDS-PAGE gels that included a 4% stacking gel composition. Initial gels were consistent with the observations by Fitts et al. (2000) who showed a single band for MHC type I and another denoting the co-migration of MHC IIa and IIx (Figure 1). To each well, 12.5 μg of sample protein was loaded and the gels were supplied with 10 mA constantly for 40 min followed by a constant voltage of 150 V for 26 h in a chilled container. Gels were then stained with Silver Stain Plus per the manufacturer's instructions (Bio-Rad, Hercules, CA). Finally, the bands for MHC isoforms were quantified using a Fluorchem SP analysis system (Alpha Innotech, San Leandro CA) to determine relative band intensity. In a minority of muscle samples, SDS-PAGE gels revealed the presence of neonatal and embryonic MHC isoforms (data not shown); the contribution of these proteins as a part of the total MHC percentage are included within the Results where appropriate.

Western blot analysis was performed for MHC type IIa due to the co-migration of the IIa/x MHC band in SDS-PAGE gels. Myofibril samples from control, HFS, and HFSR groups were separated a second time following the protocols described above. Following electrophoresis, proteins were transferred to a PDVF membrane for 3 h. PVDF membranes then were incubated in blocking solution containing 2% non-fat dry milk in PBS containing 0.1% Tween-20 (PBS-T) for 1 h. Membranes were incubated overnight at 4°C in PBS-T containing a 1:10,000 of mouse SC-71 anti-MHC IIa antibody (a gift from S. Schiaffino, University of Padova). Another membrane containing identical samples was incubated with no primary antibody and served as the control. Following incubation and 3 × 10 min rinses in PBS-T, the blots were incubated in an anti-mouse IgG-HRP (Vector, Burlingame, CA) secondary antibody in PBS-T at a 1:2000 dilution for 1 h at room temperature (RT). After incubation, the membranes were washed and placed in 10 mL of SuperSignal West Pico chemiluminescent substrate (Life Technologies) at RT. Densitometry for the resulting band intensities was performed using the FluorChem SP system. The amount of MHC type IIa was normalized by dividing the density of each band by that of a control plantaris sample from the control group that was run on the same gel.

### Western blot analysis for PGC-1α and GLUT4

For immunoblotting, 75 μg of total protein per sample were loaded into each lane for PGC-1α and GLUT4 quantification. Three controls were run simultaneously with each gel: a wide-range molecular weight marker (Bio-Rad) and total protein isolated from adult rat heart and liver. For each gel, at least one sample per group was included and grouped according to muscle type. Total protein was separated on 7.5% gels for 20 min at 80 V followed by 125 V for 1 h and then transferred to PVDF membranes for 1 h at 100 V. Membranes were blocked in 5% non-fat dry milk in PBS-T for 0.5 h then incubated with either a rabbit anti-GLUT-4 (Milipore Inc., Billerica, MA; 1:600) or a rabbit anti-PGC-1α (Abcam, Cambridge, MA; 1:1000) antibody overnight at 4°C. After washing 3 × 10 min in PBS-T, the membranes were incubated in an anti-rabbit IgG-HRP (Jackson Immunology, West Grove, PA; 1:1000) secondary antibody. Membranes were developed using Thermo Scientific Pierce ECL Western Blotting Substrate. Densitometry and quantification were completed using the ChemiDoc MP Imaging system (Bio-Rad) and Image J software (Rasband, 1997–2007).

### Statistical analyses

Comparisons were made between Con-Y, Con-O, HFS, and HFSR groups to determine if there were differences in MHC isoform content, PGC-1α, or GLUT4 associated with age, the HFS diet, and resveratrol treatment using SPSS v23. First, age comparisons between the Con-Y and Con-O were made using an unpaired, two-tailed *t*-test with equal variance and Mann Whitney tests to determine the presence of age effects. Then a one-way ANOVA and Kruskal-Wallis test were used to determine whether there were significant differences amongst the groups. The Student Newman-Keuls test was performed for pairwise comparisons of mean responses among treatment groups. Finally, to control for the co-variate effect of muscle mass and body mass, an ANCOVA was performed which confirmed a significant main effect of resveratrol on the MHC phenotypic shifts (*p* < 0.01). Significance was determined at *p* < 0.05.

## Results

### Age, body mass, and muscle masses

Mean age, body mass, and skeletal muscle (absolute and relative) masses for each group are shown in Table 2. After 2 years on the high fat/high sugar diet, the average body mass for both HFS and HFSR monkeys was 32 and 25% greater than Con-O animals, respectively (*p* < 0.05). No differences were detected for absolute muscle masses between groups. Relative Sol and Plt muscle masses were smaller in HFS and HFSR monkeys than Con-O muscles (*p* < 0.05). No differences between groups were detected for the EDL muscle.

**Table 2:** **Age, body mass, and absolute and relative muscle masses for control, HFS, and HFSR groups**.

	**Con-Y (*n* = 5)**	**Con-O (*n* = 4)**	**HFS (*n* = 8)**	**HFSR (*n* = 7)**
Age (Years)	6.8 ± 0.5	12.4 ± 0.8^*^	14.4 ± 0.7	13.7 ± 0.8
Body mass (kg)	11.2 ± 0.9	12.2 ± 1.1	16.3 ± 2.0^†^	16.3 ± 1.5^†^
**Absolute muscle mass (g)**				
Plantaris	9.7 ± 0.8	8.1 ± 0.6	9.0 ± 1.0	9.5 ± 1.0
Soleus	21.3 ± 1.8	25.3 ± 2.6	23.7 ± 2.0	20.8 ± 1.3
EDL	20.5 ± 3.3	21.7 ± 0.8	22.9 ± 2.2	23.1 ± 1.3
**Relative muscle mass (g/kg)**				
Plantaris	0.88 ± 0.06	0.68 ± 0.08	0.62 ± 0.05	0.59 ± 0.04
Soleus	1.93 ± 0.13	2.09 ± 0.14	1.55 ± 0.13^†^	1.32 ± 0.11^†^
EDL	1.84 ± 0.25	1.81 ± 0.16	1.46 ± 0.09	1.47 ± 0.13

*Values are means ± SE. Con-Y, Young controls; Con-O, older controls; HFS, hight fat/high sugar; HFSR, high fat/high sugar with resveratrol supplementation; EDL, extensor digitorum longus*.

* *Significantly different from Con-Y (P < 0.05); age comparison only performed between Con-Y and -O*.

† *Significantly different from Con-O (P < 0.05)*.

### MHC isoform mRNA and protein levels

**Figure 2:**
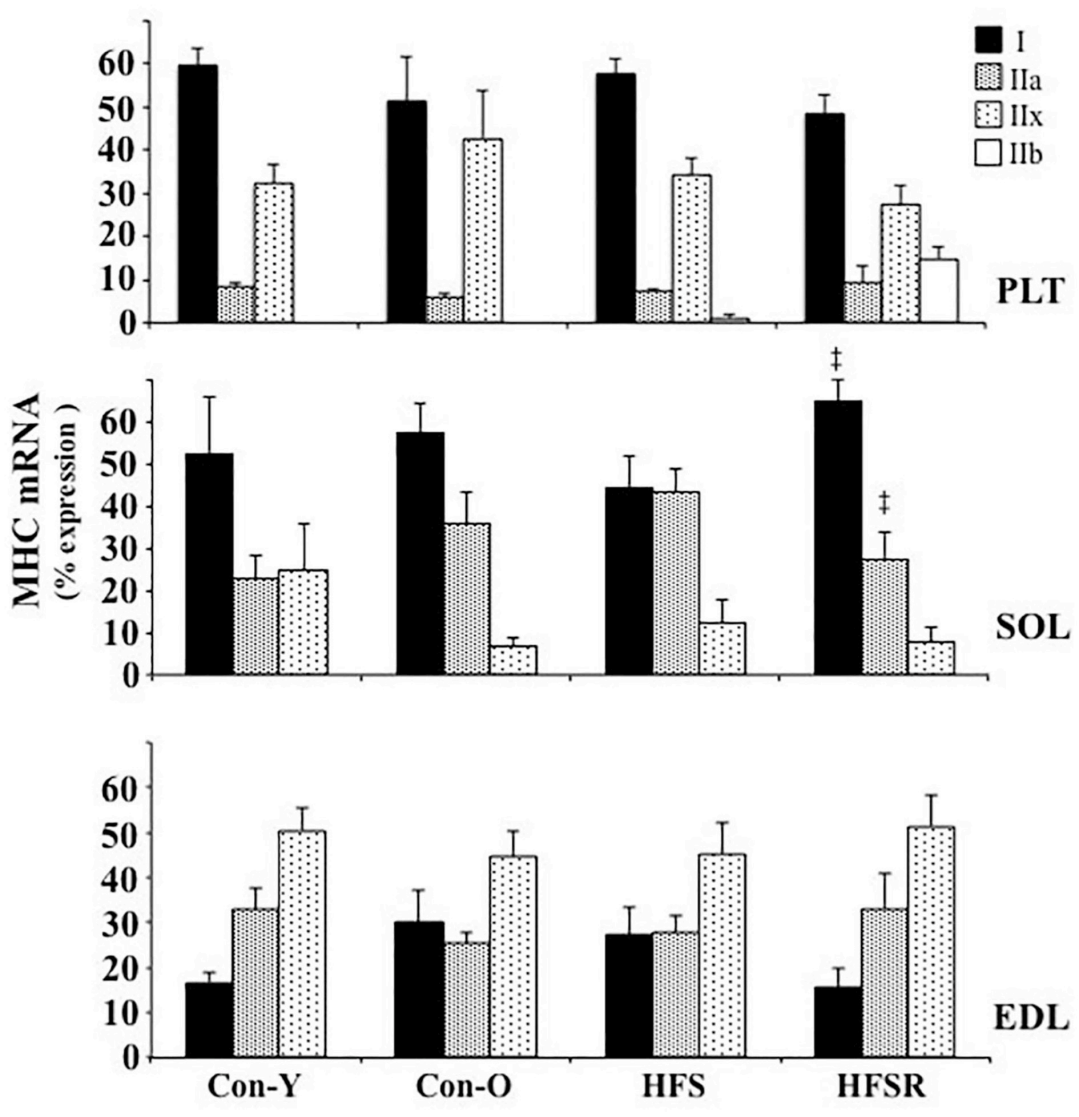
**Average changes (expressed as a percent of total MHC) in type I, IIa, IIx, and IIb mRNA as determined by quantitative real time PCR of plantaris (*top*), soleus (*middle*), and EDL (*bottom*) muscles of rhesus macaque**. Values are means ± S.E.M. Con-Y, young controls; Con-O, older controls; HFS, high fat/high sugar; HFSR, high fat/high sugar with resveratrol supplementation. ^‡^ Different from HFS (*P* < 0.05).

Quantitative real-time PCR analysis revealed muscle-specific patterns of MHC isoform mRNA within each muscle (Figure 2). There were no age-specific differences in MHC isoform mRNA expression detected in the Sol, Plt, or EDL muscles (*p* > 0.05). Plt muscles exhibited high type I and IIx isoform expression ranging between 48–60 and 27–43% for I and IIx isoforms, respectively. The MHC type IIb mRNA isoform was detected in HFS (< 1% of total MHC) and HFSR (14% of total MHC) Plt muscles and not in any other group or muscle analyzed. As expected, the MHC type I isoform was the predominant mRNA found within the Sol muscles (Figure 2). Greater amounts of MHC type I, and lower levels of type IIa mRNAs, were detected in the Sol of HFSR than HFS animals. In EDL muscles, the principal mRNA detected was MHC type IIx (Figure 2); no significant differences for any MHC mRNA levels were found between groups.

**Figure 3:**
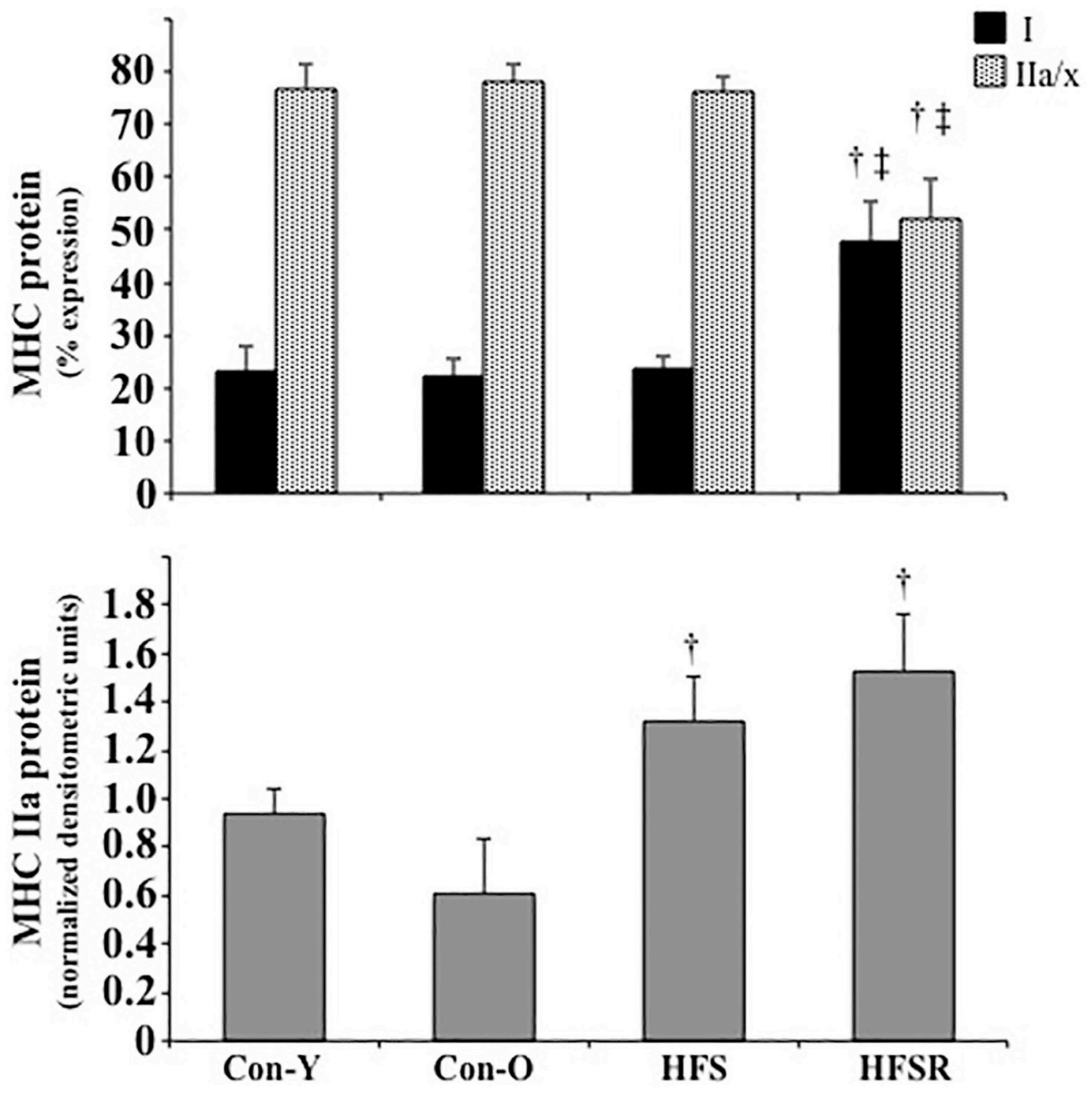
**Average changes in type I and IIa/x MHC protein isoforms as expressed as a percent of total MHC (*top*) and type IIa as determined by western analysis (*bottom*) in plantaris muscles of rhesus macaques**. Each type IIa band was normalized by dividing its density to a control plantaris sample protein band from the same gel. Values are means ± S.E.M. Con-Y, young controls; Con-O, older controls; HFS, high fat/high sugar; HFSR, high fat/high sugar with resveratrol supplementation. ^†^Different from Con-O; ^‡^Different from HFS (*P* < 0.05).

**Figure 4:**
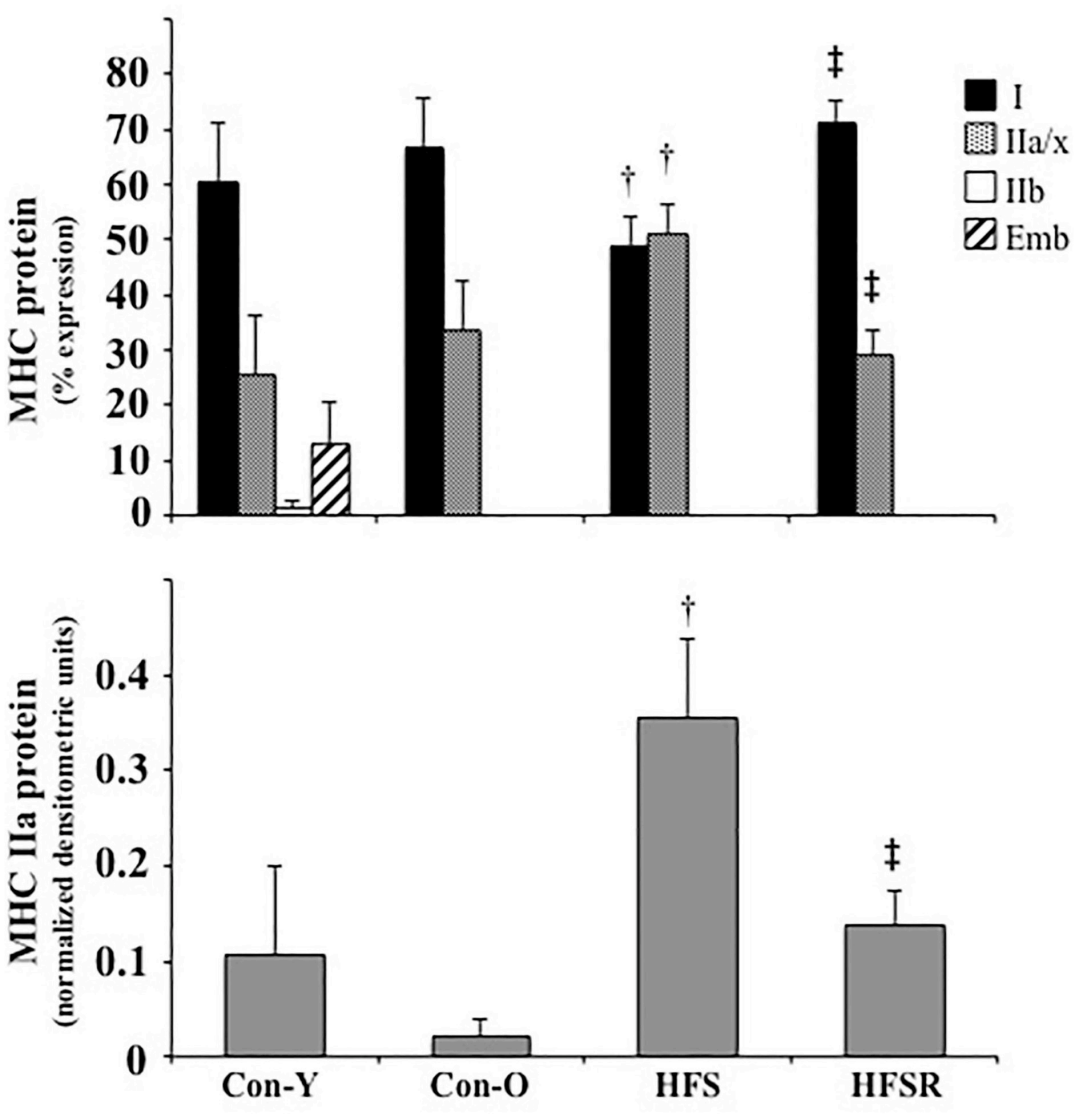
**Average changes in type I and IIa/x MHC protein isoforms as expressed as a percent of total MHC (*top*) and type IIa as determined by western analysis (*bottom*) in soleus muscles of rhesus macaques**. Small amounts of MHC type IIb and embryonic (Emb) isoforms were detected in some animals. For westerns blots, each type IIa band was normalized by dividing its density to a control plantaris sample protein band from the same gel. Values are means ± S.E.M. Con-Y, young controls; Con-O, older controls; HFS, high fat/high sugar; HFSR, high fat/high sugar with resveratrol supplementation. ^†^Different from Con-O; ^‡^Different from HFS (*P* < 0.05).

**Figure 5:**
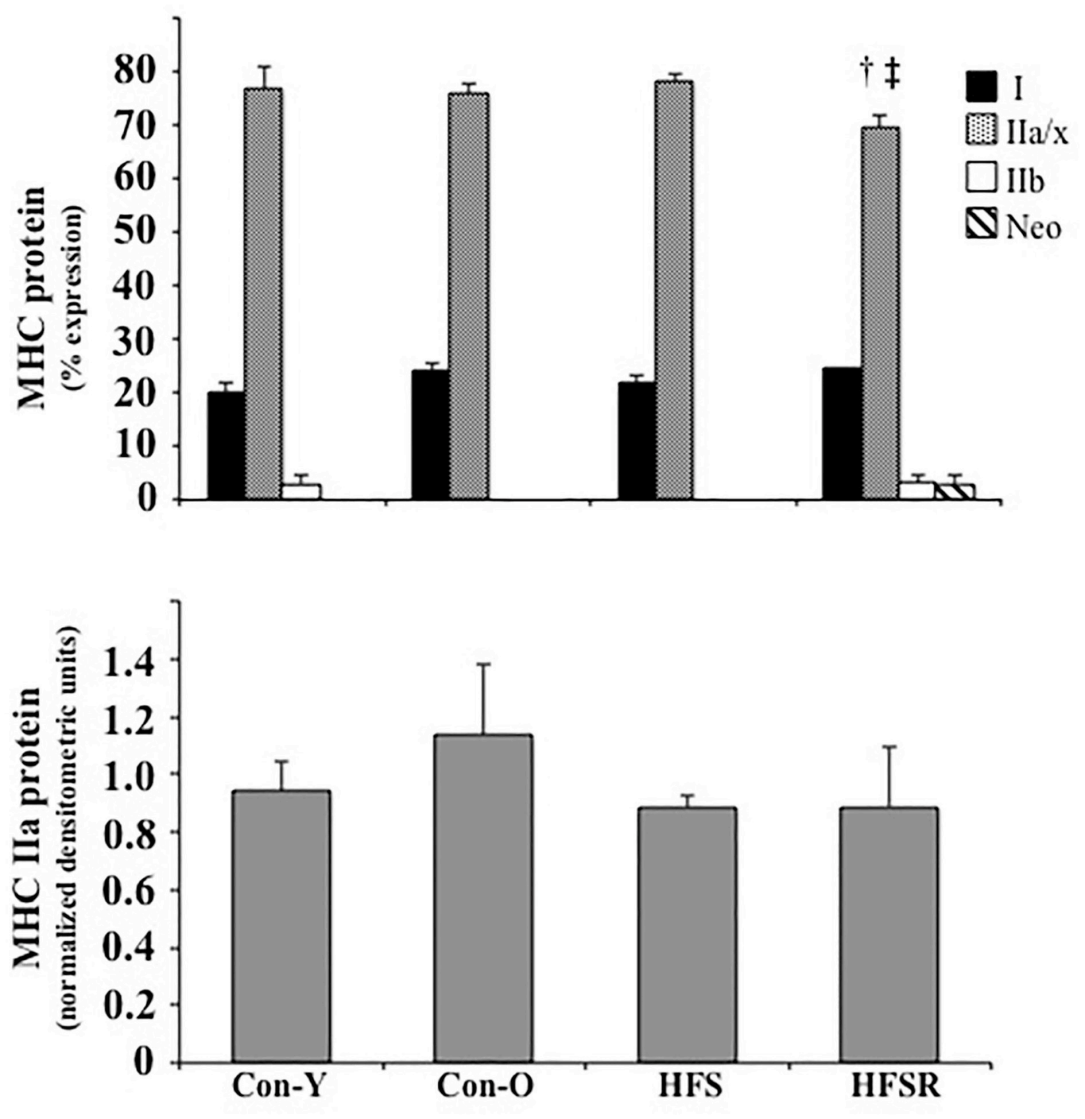
**Average changes in type I and IIa/x MHC protein isoforms as expressed as a percent of total MHC (*top*) and type IIa as determined by western analysis (*bottom*) in EDL muscles of rhesus macaques**. Small amounts of MHC type IIb and neonatal (Neo) isoforms were detected in some animals. For westerns blots, each type IIa band was normalized by dividing its density to a control plantaris sample protein band from the same gel. Values are means ± S.E.M. Con-Y, young controls; Con-O, older controls; HFS, high fat/high sugar; HFSR, high fat/high sugar with resveratrol supplementation. ^†^Different from Con-O;^‡^Different from HFS (*P* < 0.05).

MHC type I and IIa/IIx protein isoforms were the predominant isoforms detected within Plt, Sol, and EDL muscles (*top*, Figures 3–**5**). Within Sol and EDL muscles, small amounts of type IIb, embryonic, and neonatal MHC isoforms were detected in several groups; as such, only comparisons were made for MHC types I and IIa/x between groups. For each muscle, the relative expression of the MHC type IIa isoform, as determined by western analysis, is shown independently (*bottom*, Figures 3–**5**) due to the co-migration of the IIa and IIx isoforms.

In the HFSR Plt muscles, there was an increase in MHC type I protein when compared independently to Con-O and HFS groups (Figure 3); resveratrol supplementation increased type I (~25%) and decreased type IIa/x (~25%) MHC protein in the HFSR Plt muscles (*p* < 0.05). MHC type IIa western analysis identified a relatively greater amount of this isoform in HFS and HFSR groups when compared to Con-O animals (*p* < 0.05). In Sol muscles, there was a decrease in type I, and an increase in type II a/x, MHC protein of HFS monkeys when compared to HFSR and Con-O animals (Figure 4). Western analyses detected lower type IIa MHC protein levels in HFS animals when compared to Con-O and HFSR monkeys (Figure 4). Lastly, EDL muscles contained lower levels of type IIa/x MHC in HFSR animals when compared to HFS and Con-O monkeys (Figure 5). No differences were detected between groups when assessed for MHC type IIa expression (Figure 5). Resveratrol-specific influences on the MHC changes within the soleus and Plt were confirmed using regression analyses when controlling for other possible conflating factors, i.e., muscle mass, body mass, and diet (data not shown).

### PGC-1α mRNA and protein

**Figure 6:**
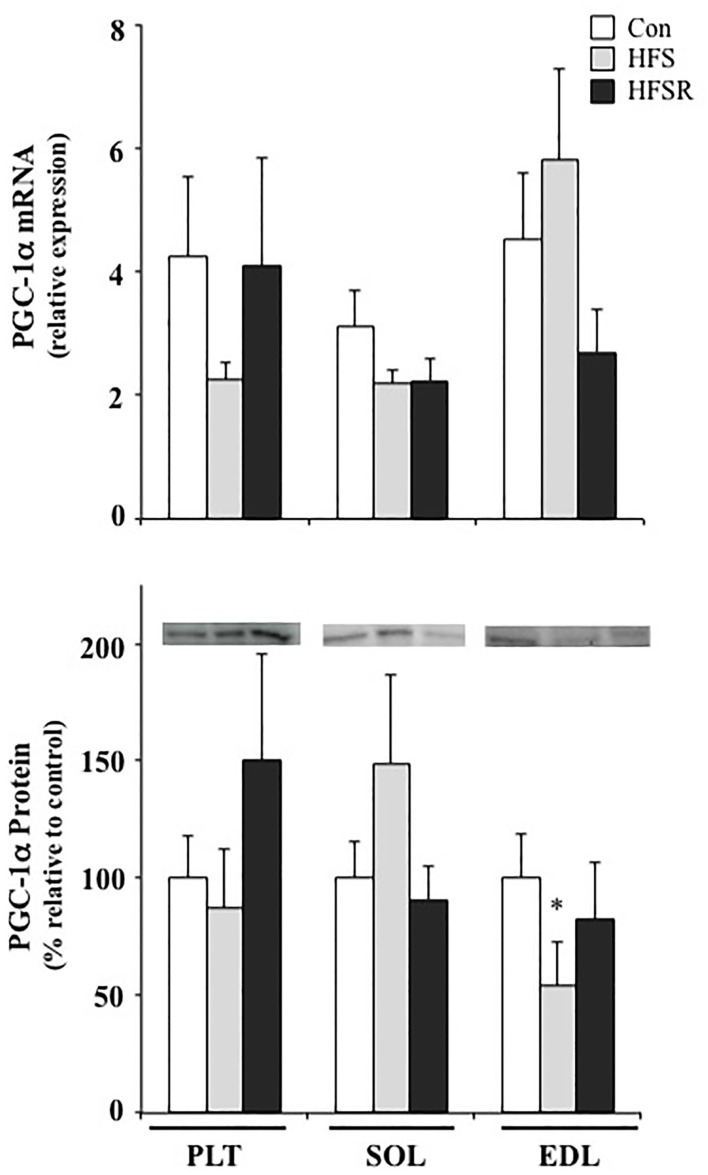
**Average changes in PGC-1α mRNA (*top*) and protein (*bottom*) in the plantaris (PLT), soleus (SOL), and ECL muscles of rhesus macaques**. Protein expression is shown as a multitude of the control value. There were no age-related statistical differences detected between young and old groups and, therefore, values were combined into a single control (Con) group. Values are means ± S.E.M. HFS, high fat/high sugar; HFSR, high fat/high sugar with resveratrol supplementation. ^*^Significantly different from Con (*P* < 0.05) using parametric *t*-tests.

PGC-1α was assessed to determine whether intramuscular levels were elevated as a result of long-term resveratrol supplementation. No age-specific differences were detected for mRNA or protein content between Con-Y and -O groups and were, therefore, combined into a single control (Con) group (Figure 6). No differences between groups were found in the Plt and Sol muscles (*p* > 0.05). In the EDL, there was lower protein detected in the HFS group when compared to the aggregated Con group (*p* < 0.05).

### GLUT4 protein

**Figure 7:**
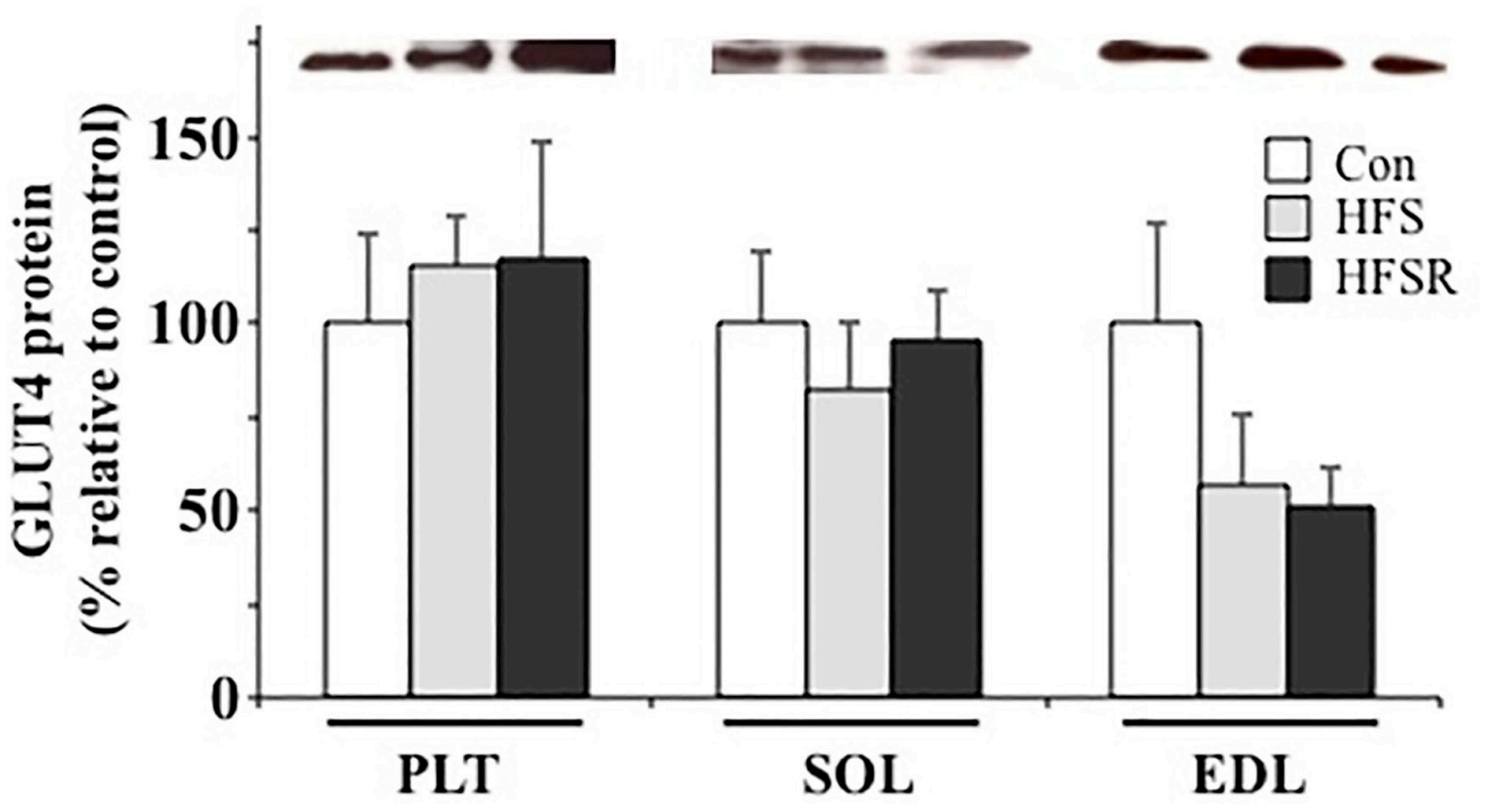
**Average changes in GLUT4 protein in the plantaris (PLT), soleus (SOL), and ECL muscles of rhesus macaques**. Protein expression is shown as a multitude of the control value. There were no age-related statistical differences detected between young and old groups and, therefore, values were combined into a single control (Con) group. Values are means ± S.E.M. HFS, high fat/high sugar; HFSR, high fat/high sugar with resveratrol supplementation.

Similarly, no age-specific differences were detected for GLUT4 protein content between Con-Y and -O groups and, therefore, were combined into a single control (Con) group (Figure 7). No statistically significant differences were detected in any muscles from any group for GLUT4 protein (Figure 7).

## Discussion

The goal of this study was to determine whether aging (Con-Y vs. -O), a long-term high fat/high sugar diet (Con-O vs. HFS), or resveratrol supplementation (HFS vs. HFSR) elicited shifts in MHC expression in three distinct hindlimb skeletal muscles from nonhuman primates. In general, we found that the soleus is the most sensitive to a high fat/sugar diet, which manifested as a higher relative proportion of type II a/x MHC than in control muscles. The plantaris and EDL muscles appeared unaffected by changes in diet alone. However, both the soleus and plantaris muscles were responsive to long-term resveratrol treatment as evident in the observed increase in the type I MHC protein, whereas the EDL remained unchanged to resveratrol treatment. These findings support the idea that there is a muscle-specific response to a high fat/sugar diet and resveratrol supplementation, which may be associated with relative daily recruitment experienced by these muscles.

### MHC mRNA and protein characteristics of the rhesus macaque hindlimb muscles

To our knowledge, this is the first study reporting MHC isoform mRNA expression in lower hindlimb muscles in this species (Figure 2), although several studies have previously investigated MHC protein isoform composition from the rhesus macaques (Fitts et al., 1998, 2000). In general, our findings show a consistent mRNA isoform distribution within muscles across experimental conditions: in the soleus muscle, type I MHC was highly expressed, whereas types I and IIx mRNA predominated in the plantaris muscle. A more even expression pattern of MHC isoform transcripts was observed within the EDL.

Consistent with mRNA expression, type I MHC protein was primarily detected in soleus muscles. The type IIa/x protein constituted the majority of MHC expression in the plantaris and EDL muscles, a pattern that was consistent with EDL but not plantaris mRNA profiles. In agreement with Baldwin et al. (2013), the differential patterns of MHC mRNA and protein particularly within the plantaris muscle allude to a high degree of post-transcriptional or pre-translational modifications occurring with MHC isoform expression.

The soleus is a highly recruited muscle through out the day because of its contributions to both locomotion and posture. The relationship between neural activity and MHC type I expression has been shown in earlier reports of other species (Pette and Vrbova, 1992; Ljubicic et al., 2005). In addition, Demirel et al. (1999) showed that sedentary rats had more type II MHC isoforms than rats that were endurance trained up to 90 min per day for 10 weeks at 75% VO_2max_, suggesting that recruitment frequency contributes, in part, to muscle phenotype. In the rhesus macaque, Hodgson et al. (2001) reported that the soleus muscle was the most active during a 24-h period of all hindlimb muscles assessed, including the tibialis anterior, medial gastrocnemius, and vastus lateralis. The high level of MHC type I expression detected in the soleus muscle of rhesus monkeys from the present study parallels the neural recruitment patterns consistently found across other animal species. However, it is unknown whether basal MHC profiles were altered due to the relative state of physical inactivity resulting from standard housing/caging of the rhesus monkeys from the present study (Talmadge, 2000).

### Age- and diet-related influences on MHC profiles

We did not observe a noticeable impact of age on MHC profiles. The age of monkeys within the Con-O, HFS, and HFSR groups was approximately 50–57% of the estimated 25-year average lifespan; as such, age may not have yet impacted the MHC profiles in these animals. Moran et al. (2005) showed that mouse EDL muscles were resistant to age-related MHC changes despite a decline in contractile force generation from 4 to 28 months of age. Short et al. (2005) found that MHC IIa and IIx mRNA in humans decreased with age by 14 and 10% per decade, respectively, but there were no age-related effects on type I MHC transcripts. In fact, this group (Short et al., 2005) reported a slight increase (3.5%) in type I MHC protein per decade of age, which parallel the findings from Larsen et al. (2012) who showed no detriments in mitochondrial respiratory capacity between young and middle-aged male subjects. A number of studies have shown fast-to-slow phenotypic transitions in aged humans with the inclusion of aerobic exercise (Konopka et al., 2011; Harber et al., 2012). The positive influence of exercise is contrasted by the findings of D'Antona et al. (2003) who found that type I MHC proportions in the vastus lateralis were drastically lowered after 3.5 months of limb immobilization in two elderly patients. Given this, the sedentary nature of the monkeys may have altered the MHC profiles reported here. Although we do not show an age effect, earlier work supports the notion that there is an age-specific influence on MHC shifts; the impact of age on MHC changes alone, however, is likely masked by the variability of muscle activity occurring across individual subjects.

Earlier work also reported that diet and obesity can influence changes in muscle phenotype. For example, comparisons between lean and obese individuals revealed a relative proportion in type II fibers within obese patients (Hickey et al., 1995; Tanner et al., 2002). The increased proportion of MHC-IIa and IIx isoforms, coupled with a decrease in type I MHC, may exacerbate the conditions of obesity if the ability to oxidize fat is reduced (Abou Mrad et al., 1992). This notion is supported by the findings of Gerrits et al. (2010) who showed that female patients who lost weight through diet restriction and physical activity interventions exhibited a greater proportion of type I fibers present within the vastus lateralis muscle when compared to age-matched obese controls. Our observed MHC shift within the soleus muscle appear consistent with previous findings that a high fat/sugar diet is associated with a reduction in the oxidative potential of skeletal muscle (Abou Mrad et al., 1992; Hickey et al., 1995; Tanner et al., 2002; Matsakas and Patel, 2009; Gerrits et al., 2010). The ~25% increase in body mass of HFS monkeys (Table 2) relative to aged-matched (Con-O) controls suggests that an impaired oxidative capacity within skeletal muscle contributed to whole-body changes. Assuming that physical activity was comparable between Con-O and HFS monkeys under caged conditions, we can reasonably conclude that diet alone has a clear influence on MHC phenotype on oxidative muscle types.

The observed MHC changes within the soleus muscle may be an indirect impact resulting from diet-related effects on the neural system. It is well established that the soleus muscle, across most species, is a relatively active muscle throughout the day (Alford et al., 1987; Hodgson et al., 2001) and is generally fatigue resistant because of its oxidative characteristics. In fact, Asmussen et al. (2003) showed that neuromuscular hyperactivity in EDL and soleus muscles of Japanese waltzing mice shifted MHC profiles to a slower phenotype. The administration of a high fat diet has been shown to slow sensory (Obrosova et al., 2007; Davidson et al., 2010; Guilford et al., 2011) and motor nerve (Obrosova et al., 2007; Guilford et al., 2011) conduction velocities. Blunted neural activity received by the soleus resulting from the high fat/sugar diet would therefore shift its MHC expression to a faster phenotype (Pette and Vrbova, 1992; Talmadge, 2000; Ljubicic et al., 2005). By comparison, the relatively inactive plantaris and EDL muscles would be affected to a lesser extent by any neural alterations resulting from a high fat/sugar diet since these muscles presumably receive little daily neural input compared to the soleus, although further work is required to discriminate the relative activity levels of the skeletal muscles within rhesus monkeys described within this study.

### Muscle-specific sensitivity to resveratrol treatment

Both soleus and plantaris muscles were responsive to long-term administration of resveratrol. In the soleus muscle of resveratrol-treated monkeys, mRNA and protein MHC isoforms were similarly distributed to that in control (Con-Y and -O) monkeys, suggesting that resveratrol may have reversed, or blunted, the slow to fast shift that was evident in the HFS group. The mechanism of action of resveratrol is not entirely understood, but several studies report a resveratrol-specific influence on calcium homeostasis or Ca^2+^-mediated signaling (Shtifman et al., 2011; Tsuda et al., 2011), suggesting that resveratrol may be modulating type I MHC expression by emulating slow Ca^2+^ dynamics. Alternatively, resveratrol may counteract the high fat/sugar changes on the neural system and normalize activity, although this is less likely given that diet-mediated changes on the plantaris muscle were not observed. Combined, these findings support the hypothesis that resveratrol treatment influences slow MHC phenotypic expression albeit in a muscle-specific manner.

Lagouge et al. (2006) reported that mice on a high fat/sugar diet with resveratrol supplementation gained significantly less weight than controls on the same diet but not receiving resveratrol. Their data suggest that resveratrol increases the basal energy expenditure and thermogenesis through the elevated expression of genes required for oxidative phosphorylation and mitochondrial biogenesis as mediated by Sirt1 de-acetylation of PGC-1α. PGC-1α signaling also has been shown to induce a fast-to-slow fiber type shift (Lin et al., 2002; Selsby et al., 2012) and increase oxidative capacity (Selsby et al., 2012) in mice. Furthermore, Michael et al. (2001) showed in cultured myotubes that ectopic expression of PGC-1α triggered GLUT4 expression, which resulted in a three-fold increase in glucose uptake in an insulin-independent fashion. We, however, did not detect any resveratrol-induced enhancements in PGC-1α or GLUT4 protein expression within soleus, plantaris, or EDL muscles (Figures 6, 7), which may be the result of a high degree of expression variability detected within the animals. Dolinsky et al. (2012) demonstrated that heart tissue from a 12-week endurance exercise + resveratrol group had elevated PGC-1α protein expression when compared to hearts from exercise-only rats, suggesting that the inclusion of physical activity augments the influence of resveratrol supplementation. It would be interesting to determine whether aerobic exercise has a similar impact on PGC-1α expression within skeletal muscle and if the fast-to-slow MHC shift induced by resveratrol treatment observed in the present study also is enhanced by aerobic exercise.

## Limitations

This impetus for conducting this study was through a collaborative opportunity; given the species used and long-term nature of the investigation, our scientific questions pertaining to skeletal muscle were generated post hoc. The study design was created well in advance of our acquisition of the muscles; as such, we acknowledge that the inclusion of a low fat (e.g., 4%) group receiving resveratrol could have increased the generalizability of this study. The sample size is an acknowledged limitation; that statistical significance was determined for MHC phenotypes in a muscle-specific manner with this limitation exemplifies the influence of diet and resveratrol in this study. Finally, it is unclear whether the animals' long-term physical inactivity had an impact on our findings and probably do not reflect activity levels of animals of this physical size and stature within a natural setting.

### Perspective

Skeletal muscles are very heterogeneous in their response to internal or external stimuli and these findings underscore how homeostasis can be modulated first by dietary changes and then by a dietary supplement in a muscle-specific manner. We found that the soleus muscle is most sensitive to alterations in diet treatment, which may be related to its relative level of daily activation when compared to the plantaris or EDL. However, both the soleus and plantaris muscles responded positively to long-term resveratrol treatment, suggesting a possible practical advantage in consuming this dietary supplement. Resveratrol supplementation may be an effective strategy in promoting the expression of the fatigue-resistant type I MHC phenotype in oxidative muscles with high degree of daily activity especially if phenotypic expression is altered as a result of long-term high fat/sugar diet. The positive changes associated with resveratrol may be accentuated with the inclusion of physical activity or aerobic exercise.

## Author contributions

Contributions to the conception or design of the work (JH, AMH, JAM, RdC, JL); Acquisition of tissue (JAM, RdC); Acquisition/analysis/interpretation—MHC protein (LN, RT, JH); Acquisition/analysis—MHC mRNA (AMH, JK, JL) and interpretation (JH); Acquisition/analysis—PGC-1α and GLUT4 (AEH) and interpretation (JH). All authors contributed to either the drafting or revising the manuscript at different stages and all have approved the final version. All authors are in agreement to be accountable for all aspects of the work in ensuring that questions related to the accuracy or integrity of any part of the work are appropriately investigated and resolved.

### Conflict of interest statement

The authors declare that the research was conducted in the absence of any commercial or financial relationships that could be construed as a potential conflict of interest.
